# Molecular investigation into the effect of carbon nanotubes interaction with CO_2_ in molecular separation using microporous polymeric membranes

**DOI:** 10.1038/s41598-020-70279-5

**Published:** 2020-08-06

**Authors:** Mahboubeh Pishnamazi, Ali Taghvaie Nakhjiri, Arezoo Sodagar Taleghani, Azam Marjani, Mashallah Rezakazemi, Saeed Shirazian

**Affiliations:** 1 Institute of Research and Development, Duy Tan University, Da Nang, 550000 Vietnam; 2 The Faculty of Pharmacy, Duy Tan University, Da Nang, 550000 Vietnam; 3 Department of Petroleum and Chemical Engineering, Science and Research Branch, Islamic Azad University, Tehran, Iran; 4 Department of Chemistry, Arak Branch, Islamic Azad University, Arak, Iran; 5 Faculty of Chemical and Materials Engineering, Shahrood University of Technology, Shahrood, Iran; 6 Department for Management of Science and Technology Development, Ton Duc Thang University, Ho Chi Minh City, Vietnam; 7 Faculty of Applied Sciences, Ton Duc Thang University, Ho Chi Minh City, Vietnam

**Keywords:** Chemistry, Theoretical chemistry, Computational chemistry, Engineering, Chemical engineering

## Abstract

The use of nanofluids has been recently of great interest to separate acidic contaminants such as CO_2_. The main objective of this research is to assess the influence of carbon nanotubes (CNTs) addition to distilled water on enhancing the CO_2_ molecular separation through a porous membrane contactor (PMC). For this aim, a comprehensive model is developed based on non-wetted and counter-current operational modes to evaluate the principal mass and momentum transport equations in tube, membrane and shell compartments of PMC. Consequently, a CFD-based axisymmetrical simulation is implemented according to finite element technique (FET) to prognosticate the results. It is found from the results that the addition of 0.1 wt% carbon nanotubes (CNTs) particles to water significantly enhances the mass transfer and consequently the CO_2_ molecular separation efficiency from 38 to 63.3%. This considerable enhancement can be justified due to the existence of two momentous phenomena including Brownian motion and Grazing effect, which enhance the mass transport of CO_2_ molecules in the PMC. Moreover, the effect of CNTs concentration, some membrane's parameters such as number of hollow fibers and porosity and also some module's design parameters including module radius and length on the CO_2_ separation performance are investigated in this paper as another highlight of the current work.

## Introduction

Sustainable anthropogenic emission of greenhouse contaminants such as CO_2_ and its pernicious influence on the atmosphere (i.e., global warming and acid rains) has been a global concern in developed and developing countries^1,2^. Globally, it is estimated that several billion tons of CO_2_ are being emitted into the atmosphere each year through burning fossil fuels^3^. Human's industrial activities are verified to be the main reason for the increase in CO_2_ amount and disturbance in atmospheric natural balance. Thus, it is vitally important to develop viable procedures for CO_2_ sequestration^4^.

There are different processes for separation and removal of CO_2_ from gas streams which need to be improved to obtain more efficient processes. Traditional techniques such as pressure swing adsorption, bubble columns, venture scrubbers, spray towers, and packed towers have recently been applied to eliminate CO_2_ contaminants from gas streams. Apart from the superb advantages of conventional methods, such techniques suffer from disparate difficulties such as high capital/operating costs, risk of liquid overflow, frothing, entrainment, and channeling^5,6^. The combination of gas absorption and membrane separation processes formed a modern technique which is known as the membrane gas separation (MGS) system. This technique is able to overcome the aforementioned drawbacks in traditional ones. Porous membrane contactors (PMC), as one of the well-known membrane separation techniques for capturing CO_2_ molecules, have been widely studied by researchers^7–10^. The gas–liquid interfacial area in PMC systems is definite and constant. Due to the laminar regime of liquid fluid inside the fibers, the hydrodynamics of the liquid side is well-known. Thus, the diffusivity of the fiber side can be simply estimated from basic principles. With no changes in the interfacial area, the gas–liquid exposure time in PMC systems can be varied independently^11,12^. Thus, owing to these characteristics, PMC can be proposed as an appropriate and promising device for sequestration of CO_2_ molecules.

It is very important to select proper absorbents in MGS systems. There are several criteria in selecting absorbents, such as chemical compatibility, viscosity, surface tension, and absorption/regeneration capability. Nanofluid, as one of the absorbents which possesses the mentioned criteria, has currently attracted tremendous attention from researchers. Nanofluids consist of nanoparticles (NPs) such as Fe_3_O_4_, Al_2_O_3_, SiO_2_, and CNTs, dispersed in base fluids like water and amine solutions^13–15^.

It has been reported that the absorption rate of the gas solutes in MGS systems can be improved by using nanofluids. For example, Peyravi et al. experimentally investigated the CO_2_ absorption enhancement by introducing various nanofluids (Fe_3_O_4_, CNTs, Al_2_O_3_, and SiO_2_) using a HFMC^16^. The authors found that Al_2_O_3_, CNTs, Fe_3_O_4_, and SiO_2_ nanoparticles could increase the absorption rate of CO_2_ by 44, 38, 25, and 3%, respectively. Golkhar et al. studied CO_2_ capture from CO_2_ and air gaseous mixture by applying water-based SiO_2_ and CNTs nanofluids through a porous polypropylene membrane^17^. They perceived that CNTs, due to their hydrophobicity and high adsorption capacity, had better separation performance compared to SiO_2_ nanoparticles and improved the CO2 adsorption rate by 40%.

Currently, the application of computational fluid dynamics (CFD) approach has been of significant interest among various researchers all over the world to solve the principal momentum/mass transfer partial differential equations (PDEs) and analyze the removal efficiency of major greenhouse contaminants like CO_2_, NO_2_, SO_2_ and H_2_S from different gaseous flows^18–23^. Pishnamazi et al. used CFD technique to evaluate the operational influence of [Bmim][BF_4_] ionic liquid addition to MEA solvent for improving the CO_2_ separation efficiency inside the porous membrane contactor (PMC). They perceived from their investigations that the addition of 30% [Bmim][BF4] ionic liquid to basic MEA absorbent resulted in a significant enhancement in the separation efficiency of CO_2_ inside the PMC from 18 to 79%^24^. Mousavian et al. theoretically studied the removal performance of CO_2_ from CO_2_/air mixture using MEA absorbent through PMC. They resulted that increase in the gas flow rate from 0.6 to 1.4 m^3^ s^−1^ decreased the CO_2_ separation from about 96 to 78%^25^.

This paper aims to implement a comprehensive modeling and its corresponding CFD-based axisymmetrical simulation to evaluate the influence of CNTs addition to distilled water on improving the molecular separation of CO_2_ molecules. Mechanistic model is developed to analyze the governing equations inside the principal domains of PMC related to mass and momentum transport processes and CFD-based axisymmetrical simulation is assembled to predict the results. As another highlight, this work aims to assess the effect of CNTs concentration, membrane's design parameters (i.e., number of hollow fibers and porosity) and also some module's design parameters (i.e., module radius/length) on the CO_2_ removal performance in order to optimize the separation process. Moreover, accurate prediction of CO_2_ sequestration percentage from CO_2_/N_2_ gaseous flow using distilled water and CNTs water-based nanofluid procedure is considered as another highlight of this investigation. Although computational simulations of membrane systems have been studied in literature, a thorough understanding the mass transfer at molecular level is required for design of this novel systems, which is addressed in the current study using a comprehensive multidimensional mechanistic model.

## Model development

**Figure 1:**
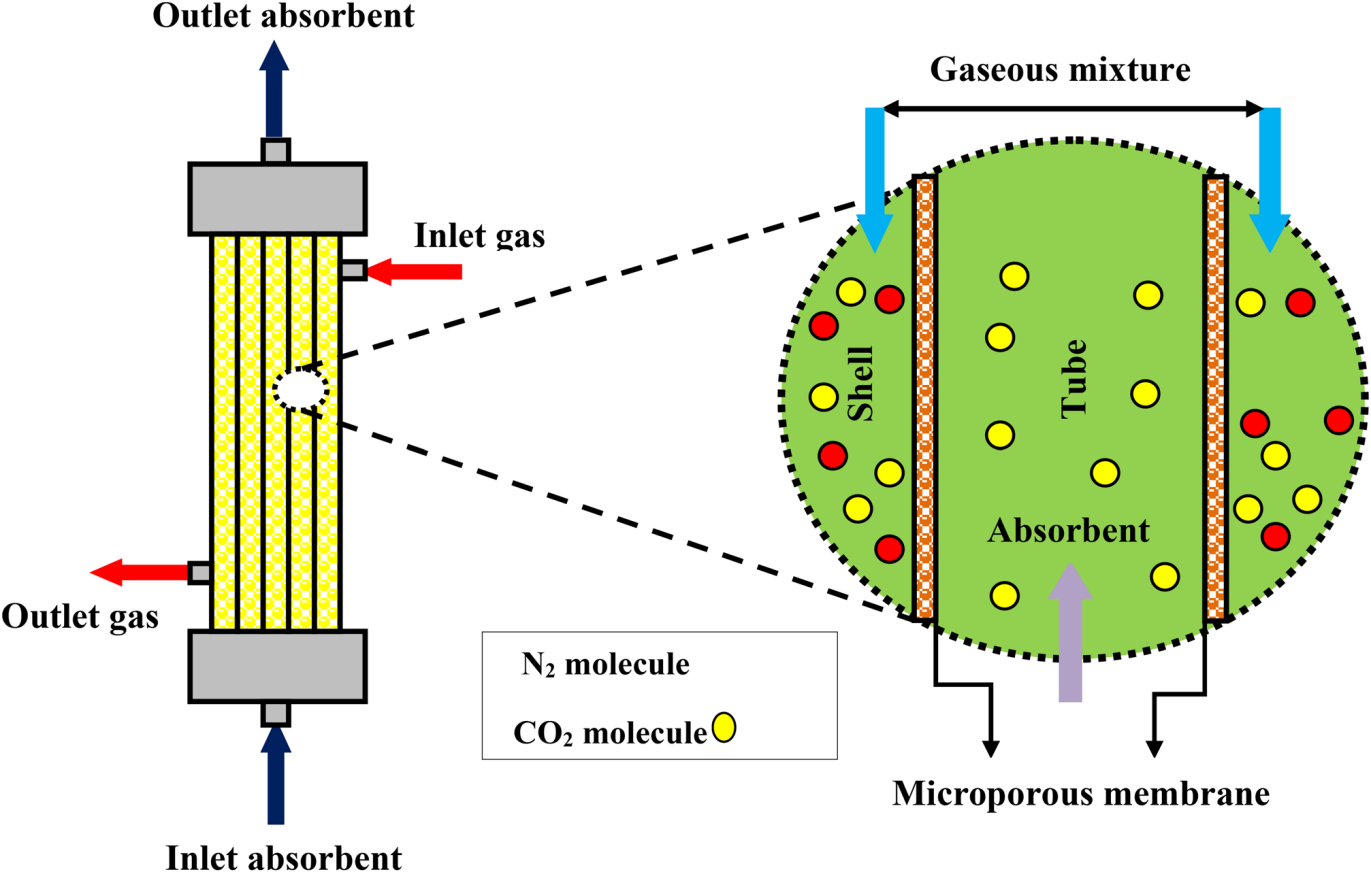
Schematic representation of CO_2_ molecular mass transport and geometrical structure of a PMC.

A mathematical modeling and its corresponding computational axisymmetrical simulation is aimed to be implemented considering a cylindrical coordinate system to numerically/computationally study the molecular removal of CO_2_ applying distilled water and CNTs water-based nanofluid as absorbents in the PMC. The schematic design of CO_2_ mass transport and geometrical structure of a PMC is presented in Fig. 1.

**Figure 2:**
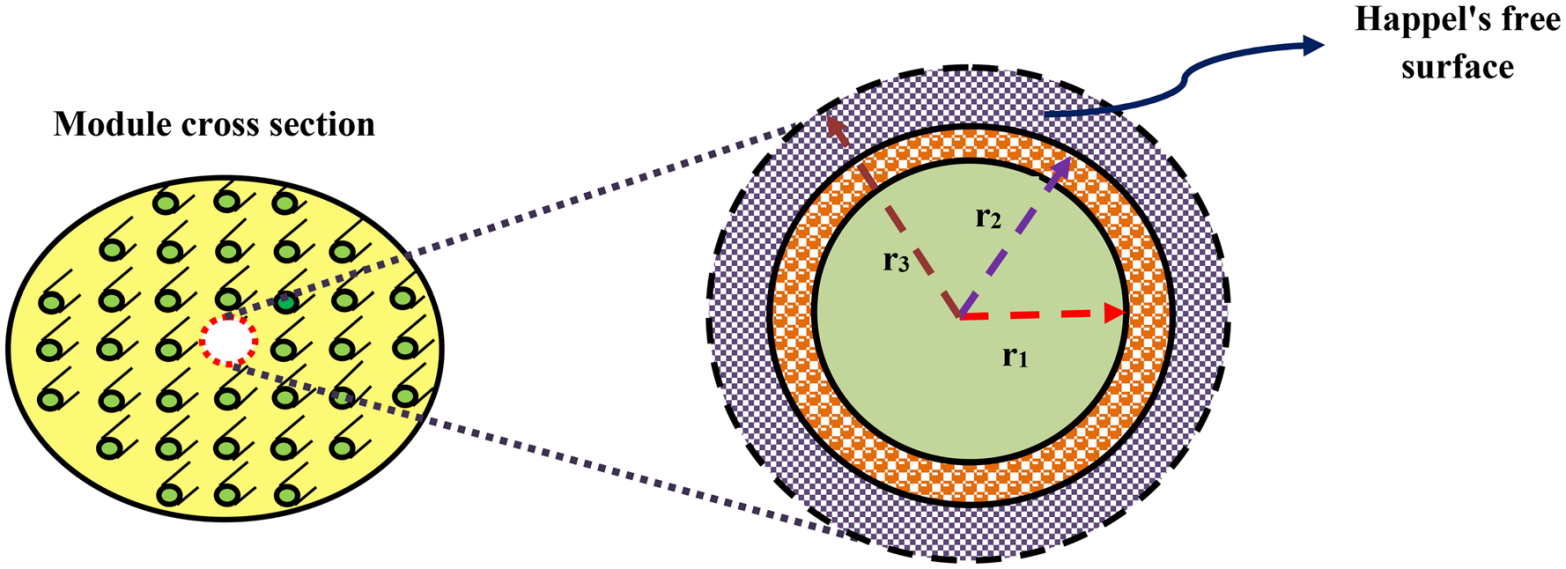
Schematic depiction of the membrane module's cross section and Happel's free surface model (HFSM).

The PMC comprised of three domains including shell, tube, and membrane. The mass transfer of CO_2_ is the consequence of the CO_2_ molecular diffusion from the gas mixture, which circulates inside the shell section (from top to bottom), to the pores of membrane wall and the chemical capture via distilled water and CNTs water-based nanofluid as absorbents flowing counter-currently through the PMC tube section (from bottom to top). Figure 2 shows the depiction of the contactor module's cross section and Happel's model. The latter deals with the influence of surrounding shell's void fraction on the fluid stream around each hollow fiber and is applied to prognosticate the assumptive effective radius (diameter) of shell (r_3_) around each fiber necessary for gas–liquid operational contact^26^.

Table 1 presents the characteristics and dimensions of CNTs existed in the CNTs water-based nanofluid considered in the simulations.

**Table 1:** The characteristics and dimensions of CNTs^16^.

Morphology	Inside diameter (nm)	Outside diameter (nm)	Length (µm)
Tubular	2.5	8	10

COMSOL software version 5.2, which functions on the basis of Finite Element Technique (FET), is used as an authentic commercial package to solve partial differential equations (PDEs) associated with the mass and momentum transport in the abovementioned sections of PMC. The UMFPACK is an eligible numerical solver among the extensive range of solvers due to its significant characteristics such as robustness and ability of solving stiff and non-stiff boundary problems. The utilized working conditions, the detailed specifications of employed PMC and physicochemical parameters of CO_2_, distilled water and CNTs water-based nanofluid are listed in Table 2.

**Table 2:** The utilized working conditions and the detailed specifications of employed PMC.

Parameter	Value	Unit	References
Inner hollow fiber radius (${r}_{1}$)	1.6 × 10^–4^	m	^16^
Outer hollow fiber radius (${r}_{2}$)	2.25 × 10^–4^	m	^16^
Module inner radius	0.01	m	^16^
Membrane porosity ($\varepsilon$)	0.5	–	^16^
Module length (*L*)	0.4	m	^16^
Total number of fibers (*n*)	400	–	^16^
Gas flow rate (*Q*_*g*_)	16	L h^−1^	^16^
Liquid flow rate (*Q*_*l*_)	7	L h^−1^	^16^
Membrane contact area	0.16	m^2^	^16^
CNTs concentration	0.1	wt%	^16^
CNTs absorption capacity	1.57 × 10^–2^	mol g^−1^	^16^
CNTs true density	2.2	g cm^−3^	^17^
Membrane tortuosity ($\tau$)	$\frac{{\left(2-\varepsilon \right)}^{2}}{\varepsilon }$	–	^27^
$\text{D}_{\text{CO}_{2}, \text{shell}}$	1.8 × 10^–5^	m^2^ s^−1^	^28^
$\text{D}_{\text{CO}_{2},\text{mem}}$	$\text{D}_{\text{CO}_{2},\text{shell}}\left(\upepsilon /\uptau \right)$	m^2^ s^−1^	^28^
$\text{D}_{\text{CO}_{2},\;\text{CNTs water-based nanofluid}}$	2.35 × 10^–6^$\exp\left(-2199/\text{T}\right)$	m^2^ s^−1^	^29^
$\text{D}_\text{CNTs water-based nanofluid,tube}$	0.5 × $\text{D}_{\text{CO}_{2}, \text{CNT water-based nanofluid}}$	m^2^ s^−1^	Estimated^30^
$\text{D}_{\text{H}_{2}\text{O-tube}}$	1.18 × 10^–6^$\exp\left(-2199/\text{T}\right)$	m^2^ s^−1^	^31^
$\text{m}_{\text{CO}_{2},\text{CNTs water-based nanofluid}}$	0.728	–	^32^
$\text{m}_{\text{CO}_{2}-\text{H}_{2}\text{O}}$	0.83	–	^33^

There are two major mechanisms for increasing the mass transport rate in the presence of nanofluids, which can be described by Brownian motion and the Grazing effect. The first mechanism is random movements of nanoparticles, which can cause an increase in the velocity and induce micro-convection around the nanoparticles and ultimately lead to mass diffusion flux enhancement as well as improvement of diffusion coefficient in the mass transfer domain^34^. The second mechanism expresses the gas adsorption in the presence of particles at the liquid–gas interface^35^. The velocity of distilled water and CNTs water-based nanofluid in the tube section is assumed to be fully developed, which may be justified by the ignorance of end effects and the particles impacts due to their low concentration. The gas flow in the shell side is described by the Happel’s model. The simplifying assumptions considered in the model development are as below^36–41^:

- Isothermal process and steady state circumstance;
- It is assumed that CNTs are spherical and homogeneous;
- The gas phase through the shell follows the ideal behavior;
- Non-wetted condition in the micropores;
- The assumption of incompressible and Newtonian fluid flow of the CNTs water-based nanofluid;
- It is assumed that radial convection can be negligible;
- Henry's law is employed to interpret the gas phase-nanofluid equilibrium;

Continuity equation for CO_2_ molecular mass transfer in the PMC's shell compartment can be attained by using Fick’s law for prediction of diffusive flux as below^42–45^:

1 $${D}_{\text{CO}_{2}, s}\left[\frac{{\partial }^{2}{C}_{\text{CO}_{2},s}}{\partial {r}^{2}}+\frac{1}{r}\frac{\partial {C}_{\text{CO}_{2},s}}{\partial r}+\frac{{\partial }^{2}{C}_{\text{CO}_{2},s}}{\partial {z}^{2}}\right]= {V}_{z,s}\frac{\partial {C}_{\text{CO}_{2},s}}{\partial z}$$

where, ${V}_{z,s}$, ${D}_{\text{CO}_{2}, s}$ and *z* are the z-direction's axial velocity in the shell part, CO_2_ diffusion coefficient inside the shell and distance along the fiber length, respectively. The gas phase velocity profile in the shell part is elucidated by the assumption of HFSM and laminar flow pattern through the following equation^40,43,46^:

2 $${V}_{z,s}=2\stackrel{-}{{V}_{s}}\left[1-{\left(\frac{{r}_{2}}{{r}_{3}}\right)}^{2}\right]\times \left[\frac{({r/{r}_{3})}^{2}-({{r}_{2}/{r}_{3})}^{2}+2\mathit{ln}\left({r}_{2}/r\right)}{3+({{r}_{2}/{r}_{3})}^{4}-4({{r}_{2}/{r}_{3})}^{2}+4\mathit{ln}\left({r}_{2}/{r}_{3}\right)}\right]$$

In Eq. (2), ${\stackrel{-}{V}}_{s}$ and ${r}_{2}$ stand for the average velocity of shell side (gas phase) and the PMC's outer fiber radius, respectively. Besides, ${r}_{3}$ describes the shell side's assumptive radius, which is computed as^28,47,48^:

3 $${r}_{3}={r}_{2}{\left(\frac{1}{1-\omega }\right)}^{0.5}$$

In Eq. (3), the packing density in the PMC is explained by $(1-\omega )$ and is computed as follows^47–49^:

4 $$1-\omega =\frac{n{r}_{2}^{2}}{{R}^{2}}$$

In Eq. (4), ${R}^{2}$ and *n* represent the module radius and the number of fibers embedded in the module, respectively. Moreover, by mixing the two previous equations (Eqs. 3 and 4), *r*_*3*_ is determineded as 0.0005 m. The utilized boundary conditions in the shell section are given as below:

5 $$at\;\; r={r}_{2}{:}\; { C}_{\text{CO}_{2},shell}={C}_{\text{CO}_{2},mem}$$

6 $$at\;\; r={r}_{3}{:}\; \partial {C}_{\text{CO}_{2},shell}/\partial r=0$$

7 $$at \;\;z=0{:}\; Convective\;flux$$

8 $$at \;\;z=L{:}\; {C}_{\text{CO}_{2},shell}={C}_{initial}$$

It is perceived that the addition of optimum amount of particles is able to increase the molecular mass transfer process of CO_2_ molecules through the PMC due to increasing the gas–liquid operational interface. The first mechanism proposed for justifying this behavior is Brownian motion. The emergence of velocity disturbance field due to particles micro-convection may enhance the diffusivity of nanofluid. For this case, no mathematical/theoretical equation exists but currently, an experimental-based relationship has been rendered in some literature. Based on these investigations, the CNTs water-based nanofluid's diffusion coefficient is derived as follows^14,50^:

9 $${D}_{nf}= {D}_{bf}\left(1+{m}_{1}{Re}^{{m}_{2}}{Sc}^{{m}_{3}}{\varnothing }^{{m}_{4}}\right)$$

In the abovementioned equation, m_1_ = 1,650, m_2_ = 0.039, m_3_ = − 1.064 and m_4_ = 0.203^14,50^. In this equation, $\varnothing$, *Sc* and *Re* are respectively denoted as volume fraction, Schmidt and Reynolds numbers for the Brownian motion of CNT. The amount of *Re* dimensionless number can be calculated using the following equation^51^:

10 $$Re= \sqrt{\frac{18KT{\rho }^{2}}{\pi {d}_{p}{\rho }_{p}{\mu }^{2}}}$$

In this equation, $K$, $T$, $\rho$, ${\rho }_{p}$, ${d}_{p}$ and $\mu$ are respectively interpreted as the Boltzmann constant, temperature, density of carrier fluid, density of CNTs particles, particles diameter, and carrier fluid's viscosity. Grazing effect (mechanism of shuttle effect in gas–liquid systems) is considered as the second principle mechanism to justify the enhancement of the CO_2_ molecular mass transfer process^17,52,53^. The Grazing effect is applied to describe the gas transport process from the liquid–gas interface to the bulk of liquid phase. The Grazing effect may be investigated by dividing the liquid phase into two separate phases including solid and liquid phases. Therefore, the mass transfer equation (continuity equation) must be derived for both liquid (distilled water) and solid (CNTs) phases. Continuity equation for CO_2_ in the solid (CNTs) is derived by the following equation^17,34,52,53^:

11 $$\varnothing {\rho }_{p}{V}_{z}\frac{\partial q}{\partial z}= {k}_{p}{a}_{p}({C}_{\text{CO}_{2},tube}-{C}_{s})$$

In Eq. (11), $${k}_{p}$$ and $${a}_{p}$$ stand for the mass transfer coefficient between CNTs and distilled water (liquid phase) and specific surface area of CNTs. The value of $$ {k}_{p}$$ is obtained via the following equation^34^:

12 $$Sh= \frac{{k}_{p}{d}_{p}}{{D}_{\text{CO}_{2}}}=2$$

Langmuir isothermal adsorption model (LIAM) is applied to calculate *q* (amount of CO_2_ molecules adsorbed by CNTs) as follows^34^:

13 $$q={q}_{m} \frac{{k}_{d}{C}_{s}}{1+{k}_{d}{C}_{s}}$$

In the above equation, ${q}_{m}$, ${k}_{d}$ and ${C}_{s}$ are respectively expressed as the highest amount of adsorption using CNTs, Langmuir constant and CO_2_ molecular concentration at the interface of liquid–solid. Application of mass balance equation for CO_2_ molecules in the tube section of PMC and considering CO_2_ adsorption on the surface of CNTs results in the appearance of C_S_. The fundamental mass transfer equation based on the steady state and non-wetted conditions for component CO_2_ molecules inside the PMC tube side is gained as below^23,28,42,44,48^:

14 $${D}_{\text{CO}_{2},tube}\left[\frac{{\partial }^{2}{C}_{\text{CO}_{2},tube}}{\partial {r}^{2}}+\frac{1}{r}\frac{\partial {C}_{\text{CO}_{2},tube}}{\partial r}+\frac{{\partial }^{2}{C}_{\text{CO}_{2},tube}}{\partial {z}^{2}}\right]= {V}_{z,tube}\frac{\partial {C}_{\text{CO}_{2},tube}}{\partial z}+\frac{{k}_{p}{a}_{p}}{1-\varnothing }({C}_{\text{CO}_{2},tube}-{C}_{s})$$

where, ${D}_{\text{CO}_{2},tube}$ stands for the diffusion coefficients of CO_2_ molecules inside the PMC's tube compartment. Moreover, ${R}_{i}$ and ${V}_{z,tube}$ state the reaction rate and axial velocity, respectively. The flow regime inside the tube is assumed to be Newtonian laminar flow. Accordingly, the axial velocity distribution can be defined by the following equation^28,43^:

15 $${V}_{z,tube}=2\overline{{V}_{t}}\left[1-{\left(\frac{r}{{r}_{1}}\right)}^{2}\right]$$

In Eq. (15), $\stackrel{-}{{V}_{t}}$, *r* and *r*_*1*_ express the average velocity in the tube side of fiber, radial direction and the inner radius of fibers, respectively. The boundary conditions employed in the tube section are interpreted as below:

16 $$at\;\; r=0{:}\; \partial {C}_{\text{CO}_{2},tube}/\partial r=0$$

17 $$at\;\; r={r}_{1}{:}\; { C}_{\text{CO}_{2},tube}={{m}_{\text{CO}_{2}}C}_{\text{CO}_{2},mem}$$

18 $$at\;\; z=0{:}\; {C}_{\text{CO}_{2},tube}=0 , \;\;\;\;{ C}_{solution,tube}= {C}_{initial }$$

19 $$at \;\;z=L{:}\; Convective \;flux$$

The continuity equation based on steady state and non-wetted operating modes inside the PMC's membrane segment is given by Eq. 20^28,38,42,43,48^. Non-wetting assumption caused that the fiber pores are only filled with the gas molecules. Hence, the principle mass transport mechanism through the membrane micropores is diffusion of CO_2_ molecules inside the gas:

20 $${D}_{\text{CO}_{2},mem}\left[\frac{{\partial }^{2}{C}_{\text{CO}_{2},mem}}{\partial {r}^{2}}+\frac{1}{r}\frac{\partial {C}_{\text{CO}_{2},mem}}{\partial r}+\frac{{\partial }^{2}{C}_{\text{CO}_{2},mem}}{\partial {z}^{2}}\right]= 0$$

In the abovementioned equation, ${C}_{\text{CO}_{2},mem}$ and ${D}_{\text{CO}_{2},mem}$ stand for the amount of CO_2_ concentration and molecular diffusion coefficient in the membrane pores, respectively. ${D}_{\text{CO}_{2},mem}$ posesses a direct relationship with membrane porosity ($\varepsilon$) and inverse relationship with membrane tortuosity ($\tau$) which can be derived as^28,40,43,44^:

21 $${D}_{\text{CO}_{2},mem}=\frac{{\varepsilon D}_{\text{CO}_{2},shell}}{\tau }$$

In Eq. 21, CO_2_ molecular diffusion coefficient in the shell of PMC is defined by ${D}_{\text{CO}_{2},shell}$. Employed boundary conditions in the membrane section are given as below:

22 $$at\;\; r={r}_{1}{:}\; { C}_{\text{CO}_{2},mem}={C}_{\text{CO}_{2},tube}/{m}_{\text{CO}_{2}}$$

23 $$at \;\;r={r}_{2}{:}\; {C}_{\text{CO}_{2},mem}={C}_{\text{CO}_{2},shell}$$

24 $$at \;\;z=0{:}\; Insulated$$

25 $$at\;\; z=L{:}\; Insulated$$

## Results and discussion

### Validation of modeling/simulation results

To verify the validity of modeling/simulation results, they are compared with existed experimental data reported in literature. Indeed, the CFD-based results of CO_2_ sequestration percentage using CNTs water-based nanofluid are compared with measured data provided by Peyravi et al. in various gas flow rates (Q_g_)^16^. The sequestration yield of CO_2_ molecules applying distilled water and CNTs water-based nanofluid is presented in the following equation, where *C* and *Q* are respectively expressed as the concentration of CO_2_ in the gas phase and volumetric gas flow rate^44,48,54^:

26 $$\text{CO}_{2} \;separation\%=100\left(\frac{{\left(Q\varphi \right)}_{inlet}-{\left(Q\varphi \right)}_{outlet}}{{\left(Q\varphi \right)}_{inlet}}\right)=100\left(1-\frac{{C}_{outlet}}{{C}_{inlet}}\right)$$

**Figure 3:**
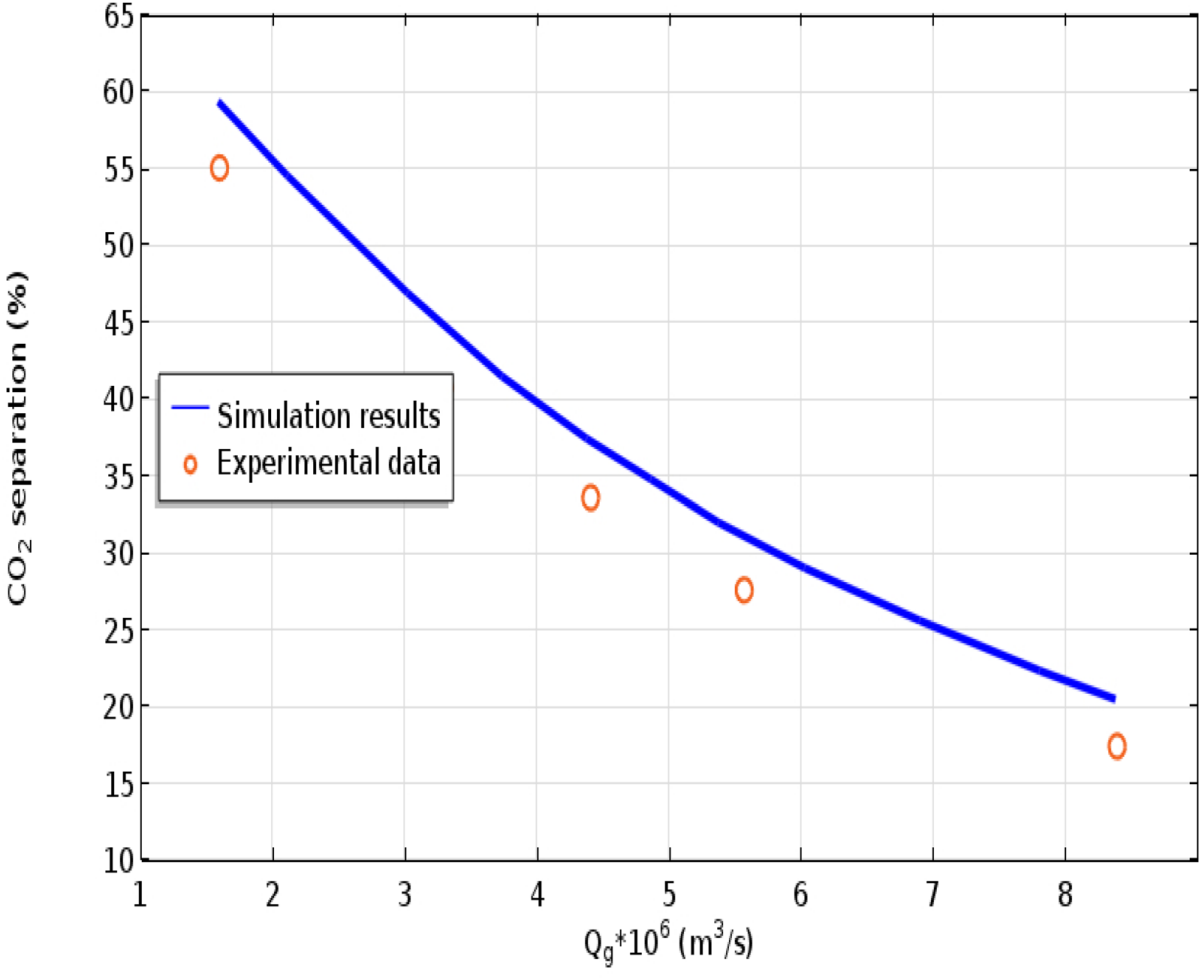
Validation of modeling/simulation results with existed experimental data provided by Peyravi et al. in various gas flow rates (Q_g_)^16^. The amount of CNTs loading = 0.1 wt% CO_2_, Inlet CO_2_ concentration = 9,900 ppm, Q_l_ = 7 L h^−1^, T = 303 K, P = 0.3 bar.

Figure 3 implies the presence of a great agreement between the measured data and modeling/simulation results for CO_2_ molecular separation percentage using CNTs water-based nanofluid with an average deviation (AD) of about 6%.

### Influence of the CNTs addition on CO_2_ concentration distribution and molecular sequestration

**Figure 4:**
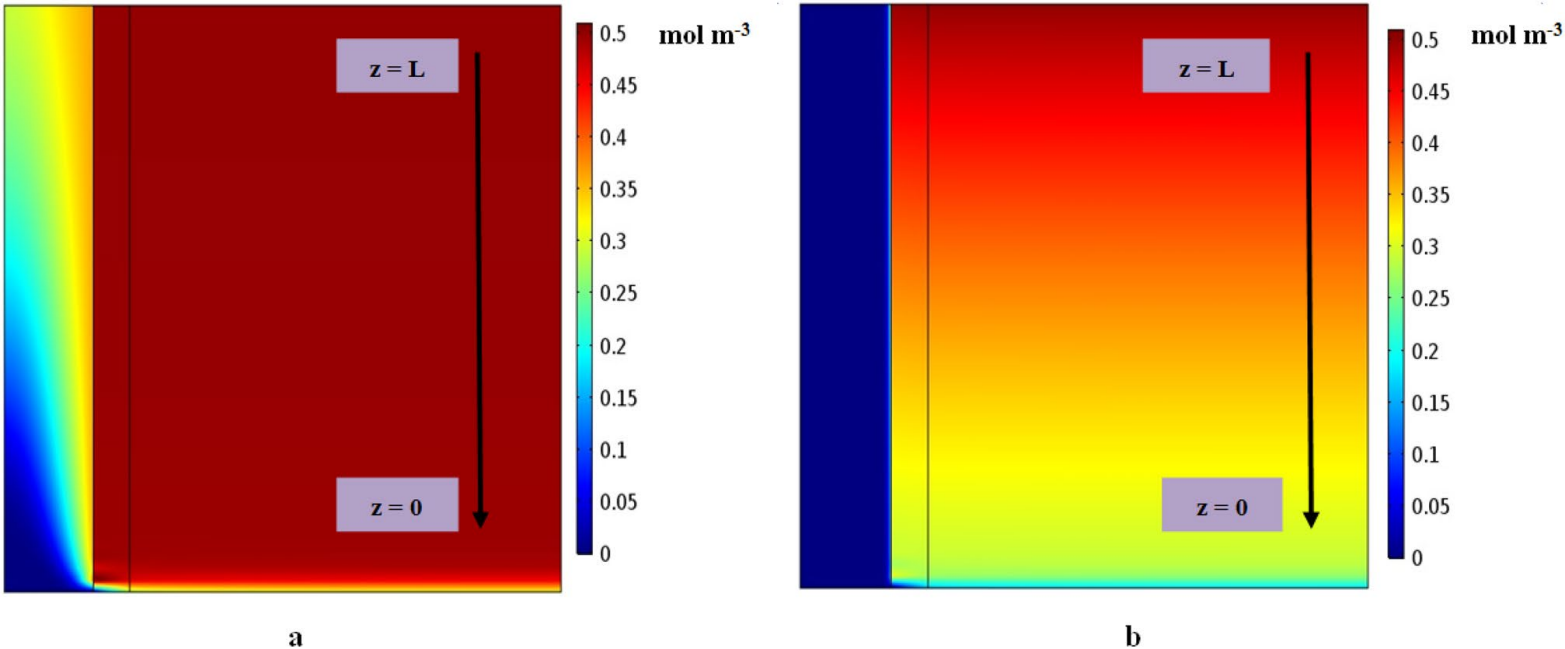
The representation of modeled surface plot of CO_2_ concentration distribution in all main domains of PMC applying (**a**) distilled water and (**b**) CNTs water-based nanofluid as absorbents.

Figure 4a,b demonstrate the modeled surface plots of CO_2_ concentration distribution in all main domains of PMC applying distilled water and CNTs water-based nanofluid as absorbents, respectively. An inlet gaseous flow containing CO_2_ and N_2_ enters the PMC from the top side (at z = L) to the bottom side (at z = 0), while the absorbents including distilled water and CNTs water-based nanofluid enter the tube side of the PMC from the bottom side (at z = 0) to the top side (at z = L) in a counter-current operational mode. Diffusion process of CO_2_ molecules takes place due to the concentration gradient from the gas mixture in the shell side through the membrane wall pores and its simultaneous removal by absorbents in the tube side. The capability of N_2_ as a carrier gas to dissolve in distilled water and CNTs water-based nanofluid is negligible compared to CO_2_. Both surface plots illustrate that the CO_2_ molecular concentration in the tube pathway of PMC is much lower than the membrane and shell side, which is attributed to the diffused CO_2_ sequestration by the distilled water and CNTs water-based nanofluid as liquid absorbents.

**Figure 5:**
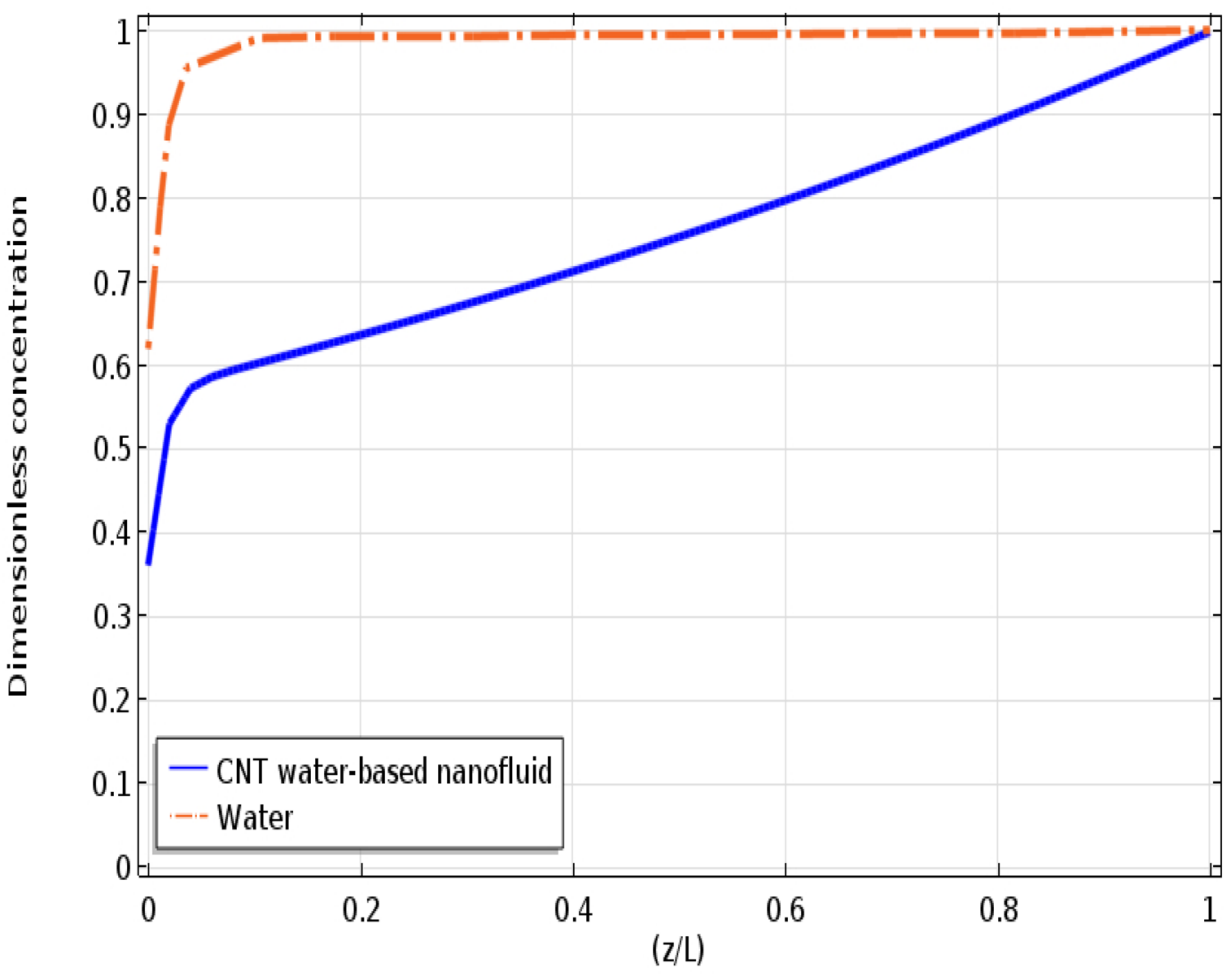
The CO_2_ dimensionless concentration applying distilled water and CNTs water-based nanofluid along the interface of shell and membrane. C_CO20_ = 0.5 mol m^−3^, Q_l_ = 7 L h^−1^, Q_G_ = 16 L h^−1^.

Figure 5 illustrates the influence of the CNTs addition to distilled water on improving the molecular sequestration of CO_2_ pollutant inside the PMC. As can be observed, addition of 0.1 wt% CNTs to water dramatically decreases the CO_2_ concentration at the outlet of shell pathway from 0.62 to 0.367, which implies a major enhancement in the molecular separation efficiency of CO_2_ acidic gas from 38 to 63.3%. This increment can be well justified due to the existence of Brownian motion in the nanoparticles that eventuates in increasing the CO_2_ molecular diffusion and consequently its mass transfer inside the CNTs water-based nanofluid.

### Influence of the CNTs concentration on the CO_2_ separation efficiency

**Figure 6:**
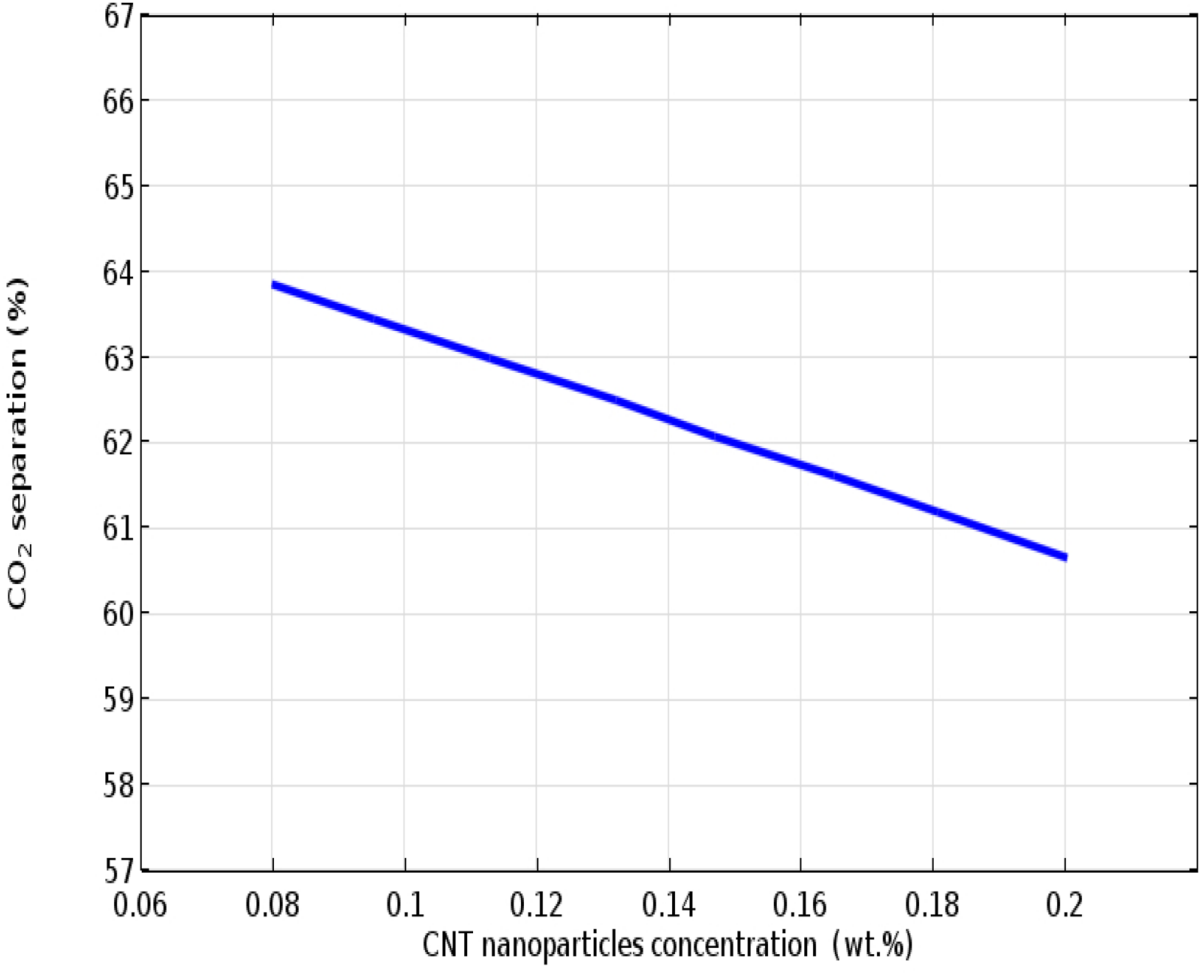
Effect of CNTs concentration on the CO_2_ separation inside the PMC.

The operational influence of CNTs concentration on the molecular separation performance of CO_2_ is rendered in Fig. 6. According to derived Eqs. (9) and (11), increment in the CNTs concentration directly influences both Brownian motion and Grazing effect. Additionally, particles concentration possesses significant effect on the particles size and nanofluids stability^55^. Following the presented explanation, enhancement of the CNTs concentration improves the agglomeration feasibility of particles. The CNTs agglomeration declines the micro-convection process in the proximity of particles, which eventuates in decreasing the effective surface area, declining mass transport rate and therefore, decreasing CO2 molecular removal performance. Additionally, the demonstrated deterioration in the CO2 removal efficiency is also associated with the decrement in the CO_2_ concentration at the solid–liquid interface that is the consequence of more particles addition. According to the Grazing effect, by declining the value of CO_2_ concentration, it's adsorption rate on the particles decreases, which possesses negative influence on the separation percentage of CO_2_ molecules. As may be observed, increase in the CNTs concentration from 0.08 to 0.2 wt% decreases the separation efficiency of CO_2_ from 65.8 to 61.7%.

### Influence of the membrane's design parameters on the CO_2_ separation efficiency

Number of porous hollow fibers and porosity are considered as the momentous membrane's design parameters, which their impacts on the separation efficiency of CO_2_ molecules are aimed to be investigated in this study. By increasing the number of porous hollow fibers in the contactor, the contact area of CO_2_/N_2_ gaseous mixture and liquid absorbing agents (distilled water and CNTs water-based nanofluid) increases, which provides more suitable opportunity for operational mass transfer process of CO_2_ molecules inside the PMC from the shell side to the fiber pores, which improves the CO_2_ molecular separation. Table 3 renders the percentage of CO_2_ molecular separation in different number of porous hollow fibers using employed distilled water and CNTs water-based nanofluid absorbing agents.

**Table 3:** Percentage of CO_2_ molecular separation in different number of porous hollow fibers.

Number of porous hollow fiber	CO_2_ molecular separation using distilled water (%)	CO_2_ separation using CNTs water-based nanofluid (%)
200	12.6	21.5
300	26.28	44.53
400	39.54	64.67
500	51.07	79.35
600	60.29	89.13

${D}_{\text{CO}_{2},mem}$ as the effective diffusion coefficient of porous membrane possesses a direct relationship with the fiber porosity. Enhancement of the fiber porosity results in improving ${D}_{\text{CO}_{2},mem}$ parameter and consequently the mass transport in the membrane side of PMC, which is the main reason of increasing the CO_2_ molecular separation percentage. Table 4 represents the percentage of CO_2_ molecular separation in different values of porosity using employed distilled water and CNTs water-based nanofluid absorbing agents.

**Table 4:** Percentage of CO_2_ molecular separation in different amount of membrane porosity.

Membrane porosity	CO_2_ molecular separation using distilled water (%)	CO_2_ separation using CNTs water-based nanofluid (%)
0.2	25.96	56.6
0.3	33.71	61.13
0.4	39.51	64.55
0.5	44.11	67.27
0.6	47.75	69.54
0.7	50.88	71.13
0.8	53.54	72.95

### Influence of the module's design parameters on the CO_2_ separation efficiency

Module radius and module length are regarded as important module-related design parameters, which their impression on the molecular separation performance of CO_2_ acidic is shown in Tables 5 and 6, respectively. As can be seen, enhancement of the module length significantly increases the duration of liquid–gas contact and also their residence time inside the PMC, which encourages the molecular separation efficiency of CO_2_. Table 5 lists the percentage of CO_2_ molecular separation in different values of module length using distilled water and CNTs water-based nanofluid absorbing agents.

**Table 5:** Percentage of CO_2_ molecular separation in different amount of module length.

Module length (m)	CO_2_ molecular separation using distilled water (%)	CO_2_ separation using CNTs water-based nanofluid (%)
0.1	14.96	25.77
0.2	24.53	42.39
0.3	32.54	54.79
0.4	39.11	64.38
0.5	44.67	71.76

**Table 6:** Percentage of CO_2_ molecular separation in different amount of module radius.

Module radius (mm)	CO_2_ molecular separation using distilled water (%)	CO_2_ separation using CNTs water-based nanofluid (%)
10	77.7	98.56
20	39.5	64.74
30	20.75	36.36
40	12	21.17

Table 6 indicates the impact of module radius on the efficiency of CO_2_ separation. According to Eq. 4, increase in the module radius (R) declines the amount of packing density ($1-\varphi )$ inside the PMC, which eventuates in decreasing the gas–liquid contact area and consequently the molecular mass transfer of CO_2_ molecules and CO_2_ molecular separation inside the PMC.

## Conclusions

In an endeavor to evaluate the influence of CNTs addition to distilled water on the removal performance of CO_2_ molecules, a mechanistic modeling and a computational 2D simulation are developed. Solution of principle equations in tube, membrane and shell compartments of PMC related to mass and momentum transport process are conducted using CFD, which operates based on finite element technique (FET). The validity of developed modeling and simulation results are corroborated by comparing them with obtained experimental data and a reasonable agreement is found with an average deviation of nearly 6%. It is understood from the simulation results that the addition of 0.1 wt% CNTs to distilled water enhances the molecular separation of CO_2_ acidic gas from 38 to 63.3%, which implies about 40% enhancement in the separation efficiency of CO_2_. This increment may be justified due to the existence of Brownian motion in the nanoparticles that eventuates in increasing the CO_2_ molecular diffusion and consequently its mass transfer inside the CNTs water-based nanofluid. It is identified from the results that the addition of CNTs is considered as a promising technology to improve the molecular separation efficiency of CO_2_ molecules inside membrane contactors.

## References

[1] Hajilary N, Shahi A, Rezakazemi M (2018). Evaluation of socio-economic factors on CO_2_ emissions in Iran: Factorial design and multivariable methods. J. Clean. Prod..

[2] Herzog, H. J. Peer Reviewed: What Future for Carbon Capture and Sequestration? (ACS Publications, 2001). 10.1021/es012307j 11348092

[3] Wang R, Li D, Liang D (2004). Modeling of CO_2_ capture by three typical amine solutions in hollow fiber membrane contactors. Chem. Eng. Process..

[4] Karoor S, Sirkar KK (1993). Gas absorption studies in microporous hollow fiber membrane modules. Ind. Eng. Chem. Res..

[5] Dindore V, Brilman DWF, Versteeg G (2005). Modelling of cross-flow membrane contactors: Mass transfer with chemical reactions. J. Membr. Sci..

[6] Zhang Z, Yan Y, Zhang L, Ju S (2014). Numerical simulation and analysis of CO_2_ removal in a polypropylene hollow fiber membrane contactor. Int. J. Chem. Eng..

[7] Tarsa ZA, Hedayat S, Rahbari-Sisakht M (2015). Fabrication and characterization of polyetherimide hollow fiber membrane contactor for carbon dioxide stripping from monoethanolamine solution. J. Membr. Sci. Res..

[8] Ghasem N, Al-Marzouqi M, Zhu L (2012). Preparation and properties of polyethersulfone hollow fiber membranes with o-xylene as an additive used in membrane contactors for CO_2_ absorption. Sep. Purif. Technol..

[9] Mansourizadeh A, Ismail AF (2011). A developed asymmetric PVDF hollow fiber membrane structure for CO_2_ absorption. Int. J. Greenhouse Gas Control.

[10] Feng C, Wang R, Zhang H, Shi L (2011). Diverse morphologies of PVDF hollow fiber membranes and their performance analysis as gas/liquid contactors. J. Appl. Polym. Sci..

[11] Ze Z, Sx J (2014). Hollow fiber membrane contactor absorption of CO_2_ from the flue gas: Review and perspective. Glob. Nest J.

[12] Mansourizadeh A, Ismail A (2009). Hollow fiber gas–liquid membrane contactors for acid gas capture: A review. J. Hazard. Mater..

[13] Choi SU, Eastman JA (1995). Enhancing thermal conductivity of fluids with nanoparticles.

[14] Nagy E, Feczkó T, Koroknai B (2007). Enhancement of oxygen mass transfer rate in the presence of nanosized particles. Chem. Eng. Sci..

[15] Rahmatmand B, Keshavarz P, Ayatollahi S (2016). Study of absorption enhancement of CO_2_ by SiO_2_, Al_2_O_3_, CNT, and Fe_3_O_4_ nanoparticles in water and amine solutions. J. Chem. Eng. Data.

[16] Peyravi A, Keshavarz P, Mowla D (2015). Experimental investigation on the absorption enhancement of CO_2_ by various nanofluids in hollow fiber membrane contactors. Energy Fuels.

[17] Golkhar A, Keshavarz P, Mowla D (2013). Investigation of CO_2_ removal by silica and CNT nanofluids in microporous hollow fiber membrane contactors. J. Membr. Sci..

[18] Al-Marzouqi MH, El-Naas MH, Marzouk SA, Al-Zarooni MA, Abdullatif N, Faiz R (2008). Modeling of CO_2_ absorption in membrane contactors. Sep. Purif. Technol..

[19] Razavi SMR, Razavi SMJ, Miri T, Shirazian S (2013). CFD simulation of CO_2_ capture from gas mixtures in nanoporous membranes by solution of 2-amino-2-methyl-1-propanol and piperazine. Int. J. Greenhouse Gas Control.

[20] Pishnamazi M (2020). Computational study on SO_2_ molecular separation applying novel EMISE ionic liquid and DMA aromatic amine solution inside microporous membranes. J. Mol. Liq..

[21] Pishnamazi M (2020). Computational fluid dynamics simulation of NO_2_ molecular sequestration from a gaseous stream using NaOH liquid absorbent through porous membrane contactors. J. Mol. Liq..

[22] Nakhjiri AT, Heydarinasab A, Bakhtiari O, Mohammadi T (2020). Numerical simulation of CO_2_/H_2_S simultaneous removal from natural gas using potassium carbonate aqueous solution in hollow fiber membrane contactor. J. Environ. Chem. Eng..

[23] Nabipour N, Babanezhad M, Taghvaie Nakhjiri A, Shirazian S (2020). Prediction of nanofluid temperature inside the cavity by integration of grid partition clustering categorization of a learning structure with the fuzzy system. ACS Omega.

[24] Pishnamazi M (2020). Computational investigation on the effect of [Bmim][BF4] ionic liquid addition to MEA alkanolamine absorbent for enhancing CO_2_ mass transfer inside membranes. J. Mol. Liq..

[25] Faravar, P., Zarei, Z. & Monjezi, M. G. Modeling and simulation absorption of CO_2_ using hollow fiber membranes (HFM) with mono-ethanol amine with computational fluid dynamics. *J. Environ. Chem. Eng.* 103946 (2020).

[26] Happel J (1959). Viscous flow relative to arrays of cylinders. AIChE J..

[27] Srisurichan S, Jiraratananon R, Fane A (2006). Mass transfer mechanisms and transport resistances in direct contact membrane distillation process. J. Membr. Sci..

[28] Faiz R, Al-Marzouqi M (2009). Mathematical modeling for the simultaneous absorption of CO_2_ and H_2_S using MEA in hollow fiber membrane contactors. J. Membr. Sci..

[29] Versteeg GF, Van Swaaij WP (1988). Solubility and diffusivity of acid gases (carbon dioxide, nitrous oxide) in aqueous alkanolamine solutions. J. Chem. Eng. Data.

[30] Ghasem N, Al-Marzouqi M (2017). Modeling and experimental study of carbon dioxide absorption in a flat sheet membrane contactor. J. Membr. Sci. Res..

[31] Hikita H, Asai S, Ishikawa H, Honda M (1977). The kinetics of reactions of carbon dioxide with monoethanolamine, diethanolamine and triethanolamine by a rapid mixing method. Chem. Eng. J..

[32] Sander R (2015). Compilation of Henry’s law constants (version 4.0) for water as solvent. Atmos. Chem. Phys.

[33] Portugal A, Magalhaes F, Mendes A (2008). Carbon dioxide absorption kinetics in potassium threonate. Chem. Eng. Sci..

[34] Sumin L, Min X, Yan S, Xiangjun D (2013). Experimental and theoretical studies of CO_2_ absorption enhancement by nano-Al_2_O_3_ and carbon nanotube particles. Chin. J. Chem. Eng..

[35] Koronaki I, Nitsas M, Vallianos CA (2016). Enhancement of carbon dioxide absorption using carbon nanotubes—A numerical approach. Appl. Therm. Eng..

[36] Mohammaddoost H, Azari A, Ansarpour M, Osfouri S (2018). Experimental investigation of CO_2_ removal from N_2_ by metal oxide nanofluids in a hollow fiber membrane contactor. Int. J. Greenhouse Gas Control.

[37] Nabipour M, Keshavarz P, Raeissi S (2017). Experimental investigation on CO2 absorption in Sulfinol-M based Fe_3_O_4_ and MWCNT nanofluids. Int. J. Refrig.

[38] Nakhjiri AT, Heydarinasab A, Bakhtiari O, Mohammadi T (2018). Influence of non-wetting, partial wetting and complete wetting modes of operation on hydrogen sulfide removal utilizing monoethanolamine absorbent in hollow fiber membrane contactor. Sustain. Environ. Res..

[39] Nakhjiri AT, Heydarinasab A (2019). Efficiency evaluation of novel liquid potassium lysinate chemical solution for CO_2_ molecular removal inside the hollow fiber membrane contactor: Comprehensive modeling and CFD simulation. J. Mol. Liq..

[40] Nakhjiri AT, Heydarinasab A, Bakhtiari O, Mohammadi T (2018). The effect of membrane pores wettability on CO_2_ removal from CO_2_/CH_4_ gaseous mixture using NaOH, MEA and TEA liquid absorbents in hollow fiber membrane contactor. Chin. J. Chem. Eng..

[41] Rezakazemi M, Darabi M, Soroush E, Mesbah M (2019). CO_2_ absorption enhancement by water-based nanofluids of CNT and SiO_2_ using hollow-fiber membrane contactor. Sep. Purif. Technol..

[42] Bird R, Stewart W, Lightfoot E (2002). Transport phenomena, 2nd edn.

[43] Nakhjiri AT, Heydarinasab A, Bakhtiari O, Mohammadi T (2018). Modeling and simulation of CO_2_ separation from CO_2_/CH_4_ gaseous mixture using potassium glycinate, potassium argininate and sodium hydroxide liquid absorbents in the hollow fiber membrane contactor. J. Environ. Chem. Eng..

[44] Nakhjiri AT, Heydarinasab A, Bakhtiari O, Mohammadi T (2018). Experimental investigation and mathematical modeling of CO_2_ sequestration from CO_2_/CH_4_ gaseous mixture using MEA and TEA aqueous absorbents through polypropylene hollow fiber membrane contactor. J. Membr. Sci..

[45] Nakhjiri AT, Heydarinasab A (2020). Efficiency evaluation of novel liquid potassium lysinate chemical solution for CO_2_ molecular removal inside the hollow fiber membrane contactor: Comprehensive modeling and CFD simulation. J. Mol. Liq..

[46] Eslami S, Mousavi SM, Danesh S, Banazadeh H (2011). Modeling and simulation of CO_2_ removal from power plant flue gas by PG solution in a hollow fiber membrane contactor. Adv. Eng. Softw..

[47] Nakhjiri AT, Heydarinasab A (2019). Computational simulation and theoretical modeling of CO_2_ separation using EDA, PZEA and PS absorbents inside the hollow fiber membrane contactor. J. Ind. Eng. Chem..

[48] Nakhjiri AT, Heydarinasab A (2020). CFD analysis of CO_2_ sequestration applying different absorbents inside the microporous PVDF hollow fiber membrane contactor. Periodica Polytech. Chem. Eng..

[49] Razavi SMR, Shirazian S, Nazemian M (2016). Numerical simulation of CO_2_ separation from gas mixtures in membrane modules: Effect of chemical absorbent. Arabi. J. Chem..

[50] Bahmanyar A, Khoobi N, Moharrer MMA, Bahmanyar H (2014). Mass transfer from nanofluid drops in a pulsed liquid–liquid extraction column. Chem. Eng. Res. Des..

[51] Prasher R, Bhattacharya P, Phelan PE (2005). Thermal conductivity of nanoscale colloidal solutions (nanofluids). Phys. Rev. Lett..

[52] Kars R, Best R, Drinkenburg A (1979). The sorption of propane in slurries of active carbon in water. Chem. Eng. J..

[53] Alper E, Wichtendahl B, Deckwer W-D (1980). Gas absorption mechanism in catalytic slurry reactors. Chem. Eng. Sci..

[54] Cussler EL (2009). Diffusion: Mass Transfer in Fluid Systems.

[55] Tantra R, Schulze P, Quincey P (2010). Effect of nanoparticle concentration on zeta-potential measurement results and reproducibility. Particuology.

